# Disparities in outcomes among patients diagnosed with cancer in proximity to an emergency department visit

**DOI:** 10.1038/s41598-022-13422-8

**Published:** 2022-06-23

**Authors:** Nicholas Pettit, Elisa Sarmiento, Jeffrey Kline

**Affiliations:** 1 Department of Emergency Medicine, Indiana University, Indianapolis, IN USA; 2 Department of Emergency Medicine, Wayne State University, 4201 St. Antoine, University Health Center – 6G, Detroit, MI 48201 USA

**Keywords:** Cancer epidemiology, Lung cancer, Oncology

## Abstract

A suspected diagnosis of cancer in the emergency department (ED) may be associated with poor outcomes, related to health disparities, however data are limited. This is a retrospective observational cohort of the Indiana State Department of Health Cancer Registry, and the Indiana Network for Patient Care. First time cancer diagnoses appearing in the registry between January 2013 and December 2017 were included. Cases identified as patients who had an ED visit in the 6 months before their cancer diagnosis; controls had no preceding ED visits. The primary outcome was mortality, comparing ED-associated mortality to non-ED-associated. 134,761 first-time cancer patients were identified, including 15,432 (11.5%) cases. The mean age was same at 65, more of the cases were Black than the controls (12.4% vs 7.4%, *P* < .0001) and more were low income (36.4%. vs 29.3%). The top 3 ED-associated cancer diagnoses were lung (18.4%), breast (8.9%), and colorectal cancers (8.9%), whereas the controls were breast (17%), lung (14.9%), and prostate cancers (10.1%). Cases observed an over three-fold higher mortality, with cumulative death rate of 32.9% for cases vs 9.0% for controls (*P* < .0001). Regression analysis predicting mortality, controlling for many confounders produced an odds ratio of 4.12 (95% CI 3.72–4.56 for cases). This study found that an ED visit within 6 months prior to the first time of ICD-coded cancer is associated with Black race, low income and an overall three-fold increased adjusted risk of death. The mortality rates for ED-associated cancers are uniformly worse for all cancer types. These data suggest that additional work is needed to reduce disparities among ED-associated cancer diagnoses.

## Introduction

Cancer is a leading cause of death worldwide^1^. The number of cancer survivors continues to grow in the face of improving therapies and earlier detection, as well as declining age-standardized incidence rates in men and stable in women^2^. Improved detection modalities is one reason for these improvements. Longitudinal outcome studies have suggested that each week added to the time to initial cancer treatment is associated with a 1–3% absolute increased risk of mortality with breast, lung, renal, and pancreatic cancers^3^. National guidelines exist for cancer screening that are relevant to emergency care. For example, the U.S. Preventive Task Force recommends yearly lung cancer screening with low-dose CT for chronic, heavy smokers, between the ages of 50–80 years old. However, only a minority of patients are appropriately screened as outpatients (4% in the setting of lung cancer), which may contribute to the burden of emergently presenting cancer^4^. Thus, there is an unmet need to define the burden of emergency department associated cancer diagnoses as to create future interventions, such as ED SBIRTs (screening, brief intervention, referral to treatment) as to either modify the risk factors for cancer (tobacco use) or referral for cancer screening^5^.

Although poorly quantified, preliminary data suggest that many patients are diagnosed with cancer during an ED visit^6^. Because patients who have cancer diagnosed during ED presentations may have multiple inequities in care (lack of insurance, no primary care, and possibly higher smoking rates), their outcomes may be worse. However, since the literature is sparse, and mostly retrospective, little is absolutely known about the outcomes of patients with emergently diagnosed cancer.

In this work, we use data linkage and in a retrospective observational cohort methodology to characterize the demographics, phenotypes, and outcomes of patients that have ED-associated cancer diagnoses. We used a statewide database to examine the rate of recent visit–defined arbitrarily as within six months–to an ED in patients with newly diagnosed cancer with the inference that the recent visit was the touchpoint where the initial clinical information suggested a possible new cancer diagnosis. We hypothesized that ED-associated cancer diagnoses will suffer higher mortality as compared to non-ED-associated cancer diagnoses.

## Methods

### Patients

This is a retrospective observational cohort study from patients with cancer in the state of Indiana, located in the USA. Data was obtained from Indiana State Department of Health (ISDH) Cancer Registry, the Indiana Network for Patient Care (INPC), supplemented with data from the electronic health records (EHR) of two major hospitals in the greater Indianapolis area. Data was curated and provided by The Regenstrief Institute which includes 47 hospitals and 3 insurance institutions^7^.

Patients with their first cancer diagnosis between January 2013 and December 2017 were identified using the ISDH Cancer Registry. To determine presence or absence of a recent ED visit, clinical features and outcomes, patients in the ISDH Cancer registry were co-identified in the INPC research database, which represents one of the largest health information exchanges in the country. Cases were defined as patients with any ED visits in the 6 months before their cancer diagnosis were and controls were all others (irrespective of reason for visit). Six months was chosen as the studied time frame, in comparison to the previously published 30-day definition of an emergency presentation from UK-based data, as many patients in the USA wait upwards of 90 days or more from first presentation to treatment^8,9^.

The index date for all patients was set as the date of initial cancer diagnosis according to the first posted cancer-defining ICD code. The ICD Oncology Topography codes, the cancer primary site, as well as patient zip code and insurance used at the time of diagnosis were extracted from the ISDH Cancer Registry.

Using unique identifiers for data linkage, demographics and clinical data, comorbid ICD codes were downloaded from the INPC and supplemented with ISDH Cancer Registry data. The Charlson Comorbidity Index (CCI) at time of index was then calculated using diagnoses within the year prior to index^10^. Social history (tobacco use, drug use, and alcohol use) was also identified using ICD codes. Tobacco use was extracted from local hospital electronic health records. Vital status and patient mortality was extracted using Social Security Administration (SSA) death data linked to the INPC and calculated in days from the index ED visit. Where SSA data was missing, death data was extracted from the INPC and the ISDH Cancer Registry.

### Statistics

Patient demographics and characteristics were compared between those patients who did not have an ED visit within six months prior to cancer diagnosis (controls) and those patients who did (cases). Missing data were excluded from analysis. Categorical variables were presented as frequencies, and continuous variables were presented as medians (with interquartile ranges). To test for differences between groups, the Chi-square test or 95% confidence interval for differences in proportions were used for bivariate variables, and the Wilcoxon test for continuous variables. Adjusted odds ratios from logistic regression containing the following dependent variables (age, gender, race, SES, drug use, alcohol use, tobacco use, and the Charlson comorbidity index)was used to determine the association of ED diagnosis (cases) with minority status, low SES status and death compared with non-ED diagnosis (controls). A generalized estimating equation using the GENMOD procedure was used to adjust for clustering at the city level via zip code which was the estimate of SES. We performed statistical analyses using SAS version 9.4 (SAS Institute, Cary, NC).

### Ethics approval

This work was approved under exempt status by the Indiana University Institutional Review Board (IRB #1,808,879,348). De-identified data was used for this study, and informed consent to participate was waived by the IRB. All methods were carried out in accordance with relevant guidelines and regulations.

## Results

### Comparing the two populations

Table 1 gives the demographics and characteristics of the patients involved in this study, separating patients into controls, those with no-associated ED visits in prior 6 months and compares those to case patients with an associated ED visit within 6 months from the time of cancer diagnosis. In total, 134,761 patients were identified to be diagnosed with cancer within the Indiana Cancer Registry, with 15,432 (11.5%) cases. First time cancer diagnoses were identified during the 2013–2017 study period, but mortality was included up to 2019.

**Table 1:** Characteristics of the study population.

	Total N = 134,761 N (percent)	Controls (No ED visit within six months prior) N = 119,329 N (total cohort percent*, column percent^#^)	Cases (ED visit within six months prior) N = 15,432 N (total cohort percent, column percent)
**Gender**			
Female	70,590 (52.4)	62,614 (46.5, 52.5)	7,976 (5.9, 51.7)
Male	64,165 (47.6)	56,709 (42.1, 47.5)	7,456 (5.5, 48.3)
Unknown	6 (0.0)	6 (0.0, 0.0)	0 (0.0, 0.0)
**Age at diagnosis,** Median (IQR)	65.0 (55.0–74.0)	65.0 (56.0–73.0)	65.0 (54.0–75.0)
**Race**			
American Indian or Alaska Native	89 (0.1)	74 (0.1, 0.1)	15 (0.0, 0.1)
Asian	953 (0.7)	874 (0.7, 0.7)	79 (0.1, 0.5)
Black	10,770 (7.9)	8,858 (6.6, 7.4)	1,912 (1.4, 12.4)
Native Hawaiian or Other Pacific Islander	31 (0.0)	28 (0.0, 0.0)	3 (0.0, 0.0)
Other	79 (0.1)	77 (0.1, 0.1)	2 (0.0, 0.0)
Unknown	1,185 (0.9)	1,098 (0.8, 0.9)	87 (0.1, 0.6)
White	121,654 (90.2)	108,320 (80.4, 90.8)	13,334 (9.9, 86.4)
**Drug use**			
No	133,242 (98.9)	118,324 (87.8, 99.2)	14,918 (11.1, 96.7)
Yes	1,519 (1.1)	1,005 (0.8, 0.8)	514 (0.4, 3.3)
**Alcohol use**			
No	131,046 (97.24)	116,579 (86.51, 97.70)	14,467 (10.7, 93.8)
Yes	3,715 (2.76)	2,750 (2.04, 2.30)	965 (0.7, 6.3)
**tobacco use**			
No	98,521 (73.1)	89,309 (66.3, 74.8)	9,212 (6.84, 59.69)
Yes	36,240 (26.9)	30,020 (22.2, 25.2)	6,220 (4.62, 40.31)
**Vital status**			
Alive	118,910 (88.2)	108,548 (80.6, 90.9)	10,362 (7.7, 67.2)
Deceased	15,851 (11.8)	10,781 (8.0, 9.0)	5,070 (11.8, 32.9)
**CCI,** Median (IQR, Range)	2.0 (1.0–3.0)	2.0 (1.0–3.0, 1.0–19.0)	2.0 (1.0–4.0, 1.0–17.0)

Abbreviations: ED-emergency department, CCI-Charlson comorbidity index.

Pertinent differences included the finding that African Americans comprised a significantly greater proportion of cases (12.4% versus 7.4, 95% CI for difference of 5.0% = 2.3 to 6.4%). Furthermore, cases had higher rates of drug, alcohol, and tobacco use than controls at 3.3% vs 0.8% (95% CI for 2.5% = 0.20–0.25) for drug use, 6.3% vs 2.3% for alcohol use (95% CI for 4.0% = 0.14–0.16), and 40.3% vs 25.2% for tobacco use (95% CI for 15.1% = 0.07–0.08). In terms of primary outcome, cases had a significantly higher mortality rate of 32.8% versus 9.0% (95% CI for 23.8% = 0.23–0.24) within a median 116 days for cases and 268 days for controls.

### Top cancers per cohort

Table 2 lists the top 10 malignancies for both cohorts in order of decreasing frequency. The values within each column for frequencies gives two percentages, with the first percentage the overall percentage and the second being the percentage just within controls or cases. For controls, the top 3 cancers in decreasing frequency are breast (18.1%), lung (14.4%), and prostate (10.1%). Comparatively the most frequently ED-associated cancers were lung (18.4%), breast (8.9%), and colon/rectal (8.9%).

**Table 2:** Frequency of cancers among controls and cases.

Cancer type*	Total N = 134,761 N (percent)	Controls (No ED visit within six months prior) N = 119,329 N (total cohort percent^&^, column percent^#^)	Cancer type*	Cases (ED visit within six months prior) N = 15,432 N (total cohort percent, column percent)
Breast	22,978 (17.05)	21,606 (16.03, 18.11)	Bronchus/Lung	2,838 (2.11, 18.39)
Bronchus/lung	20,023 (14.86)	17,185 (12.75, 14.40)	Breast	1,372 (1.02, 8.89)
Prostate gland	13,593 (10.09)	12,677 (9.41, 10.62)	Colon/Rectum	1,369 (1.0, 8.87)
Colon/rectum	11,887 (8.82)	10,518 (7.80, 8.81)	Hematopoietic and Reticuloendothelial Systems	1,066 (0.79, 6.91)
Hematopoietic and Reticuloendothelial Systems	7,360 (5.46)	6,294 (4.67, 5.27)	Prostate Gland	916 (0.68, 5.94)
Bladder	5,887 (4.37)	5,068 (3.76, 4.25)	Bladder	819 (0.61, 5.31)
Kidney	5,015 (3.72)	4,211 (3.12, 3.53)	Kidney	804 (0.60, 5.21)
Corpus uteri	4,668 (3.46)	4,197 (3.11, 3.52)	Pancreas	664 (0.48, 4.17)
Lymph nodes	4,264 (3.16)	3,662 (2.72, 3.07)	Lymph Nodes	602 (0.45, 3.90)
Pancreas	3,914 (2.90)	3,270 (2.43, 2.74)	Liver And Intrahepatic Bile Ducts	475 (0.35, 3.1)

*Displaying top 10. ^&^total cohort percent is the column value divided by the N total (137,761), ^#^column percent is the column value divided by the column’s total N.

### Comparing mortality between cancers

**Figure 1:**
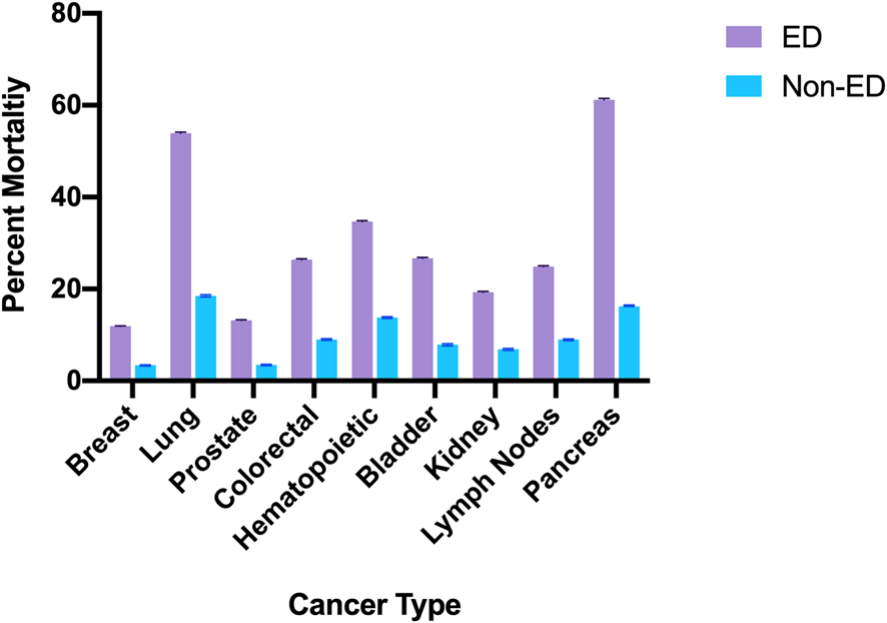
Bar graph comparing mortality among nine cancers. Bar graph comparing mortality percentages from cases compared to controls for the top 9 most common cancers, over 5-year study period, error bars representing 95% CI.

**Figure 2:**
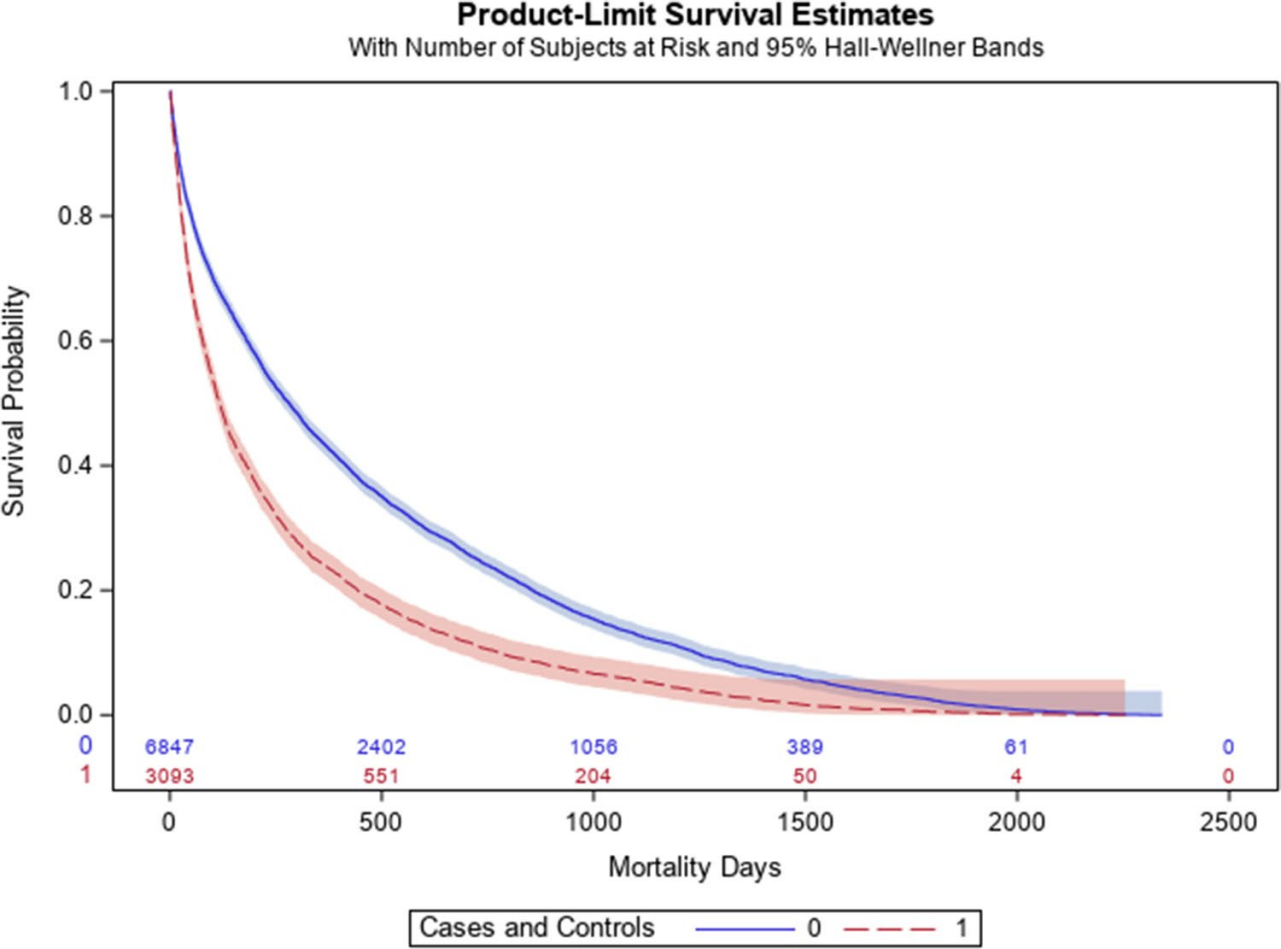
Survival curve for cases vs. controls. Kaplan Meier survival curve comparing days lived from cases (blue line) to controls (red line).

Figure 1 (S1 Table) compares the cumulative mortality rates for 9 cancer diagnoses during the 5-year study period, (since the 10th cancer in each was different between the two). The percentage of mortality for all nine cancer types were consistently higher in cases compared with controls. A concerning 45% relative difference exists between cases and controls for pancreatic cancer. Figure 2 shows Kaplan Meier survival curves for these nine cancers and demonstrates a consistently worse survival for cases versus controls up until 5 years of follow-up (*P* < 0.001 log-rank statistic).

### Socioeconomic status and racial outcomes

Table 3 compares SES data between cases and controls, and was assigned by zip code into 5 different categories, namely below/near poverty, low income, middle class, upper middle class, and highest tax bracket^11,12^. Among cases, 36.9% were low income or below poverty level, compared with 29.5% in the controls (95% CI for difference of 7.4% = 0.03–0.04). Supplemental Table 3 breaks down the mortality among cases and controls based upon SES. Similar to the data presented in Figs. 1 and 2, mortality among cases consistently worse; overall, 36.3% of low-income cases having died in the study period, whereas only 11% low-income controls died.

**Table 3:** Socioeconomic status for controls vs. cases.

Total N = 134,761	Controls (No ED visit within six months prior) N = 118,599 N (total cohort percent^&^, column percent^#^)	Cases (ED visit within six months prior) N = 15,344 N (total cohort percent, column percent)
Socioeconomic status*		
Below or near poverty level	214 (0.16, 0.18)	31 (0.02, 0.20)
Low income	34,794 (25.98, 29.34)	5,583 (4.17, 36.39)
Middle class	83,568 (62.39, 70.46)	9,729 (7.26, 63.41)
Upper middle class	10 (0.01, 0.01)	0 (0, 0)
Highest tax brackets	13 (0.01, 0.01)	1 (0, 0.01)

*See text for description of socioeconomic class, **P < 0.001 estimated by Chi-square, ^&^total cohort percent is the column value divided by the N total (137,761), ^#^column percent is the column value divided by the column’s total N.

Black race consistently worsened cancer prognosis among cases (S2 Table). For breast cancer, mortality rate was 12.1% for Black patients who were ED cases, versus a mortality rate of 5.5% for Black patients who were in the control group (95% CI for 6.6% difference = 0.01–0.04). Black patients comprised 18.2% of the breast cancer patients among cases versus 8.0% of controls (95% CI for 10.1% difference = 0.06–0.09). These trends continued for lung cancer, with a mortality rate of 58.7% among Black patients in case group, compared with a mortality rate of 22.4% among the control group.

### Controlling for confounders

Table 4 shows the result of logistic regression to examine the effect of case or control status on mortality while controlling for confounding variables. The equation was designed to control for clustering by city (via zip code) and also for seven other potential independent predictor variables (age, gender, race, SES, drug use, alcohol use, tobacco use, and the CCI) and found cases had an increased risk of death over controls with an adjusted odds ratio of 4.12 (95% CI 3.72–4.56). After these corrections, the lower limit of the 95% CI for mortality consistently remained above 1.0 for case versus control status for all cancer.

**Table 4:** Mortality risk based upon recent emergency department (ED) visit, minority and socioeconomic status.

Recent ED visit	Odds ratio (95% CI)*
Seen 6 months prior (unadjusted)	4.93 (4.40–5.51)
Seen 6 months prior (adjusted**)	4.12 (3.72–4.56)

Minority status (non-whites)	Odds Ratio (95% CI)*
Seen 6 months prior (unadjusted)	1.55 (1.24–1.94)
Seen 6 months prior (adjusted**)	0.97 (0.96–0.99)

Low socioeconomic status	Odds Ratio (95% CI)*
Seen 6 months prior (unadjusted)	1.37 (1.01–1.87)
Seen 6 months prior (adjusted**)	1.19 (0.94–1.50)

*Odds of death comparing cases with controls using logistic regression.

**Adjusted for age, gender, race, SES, drug use, alcohol use, tobacco use, & Charlson Score.

## Discussion

Our findings show uniformly worsened outcomes of all patients during a period of five years, especially in minority and low-income patients, diagnosed with new cancer diagnosed in temporal proximity to an ED visit. These data infer that patients with undiagnosed cancer who rely on the ED as a source of primary medical care will have a worsened outcome compared with patients who have organized medical care^6^. Prior work from the United Kingdom’s “Routes-to-Diagnosis” conducted by the Public Health England, suggested 23% of newly diagnosed cancer patients presented emergently and survival rates were much lower for those emergent presenters, which is similar to what we discovered in this work^13^. For example, in a recent cross-sectional population-based study from 14 jurisdictions in six countries demonstrated that 24.0–42.5% of patients were defined as emergency presentations of cancer^8^. Emergency presentation, which is associated with various cancer types, older age, and advanced stage at diagnosis, strongly predicts worse prognosis and probably contributes to international differences in cancer survival^8^. Notably absent from this recent meta-analysis of 857,068 patients across 6 countries are patients from the USA.

In the US, a study from Michigan, lung and colorectal cancer patients with ED-associated cancer diagnosis had more advanced staged cancer and were more likely to be Black than those diagnosed without a recent ED visit, but no data on mortality were presented^14^. In this study, the authors chose a conservative approach by defining an ED-diagnosed cancer as one that had an ED visit in the month of or month before the diagnosis of cancer, however, prior to this publication there is no precedent for defining an ED-associated cancer. Instead, we chose 6 months as our conservative time point as to define ED-associated cancer, as there is substantial evidence that ambulatory follow-up compliance after ED discharge is poor, and has been estimated to be between 26 and 56% depending on the ED population^15^. Additionally, there is some evidence that suggests almost one third of patients with new onset cancer experience delays of over 90 days, and thus there is no precedent for establishing the optimal time from ED visit to cancer diagnosis^9^. Among 9,470,626 ED visits among Medicare beneficiaries aged 65 and older, nearly 30% lacked ambulatory follow-up at 30 days, with lower rates of ambulatory follow-up observed among those of Black race and seen in rural EDs^16^. Further, a study from Western Australia demonstrated that of 1358 people with incident breast, prostate, colorectal, and lung cancers the diagnostic interval from symptom to diagnosis ranged from 92–108 days, further suggesting the time to cancer diagnosis can exceed several months^17^. Rural health disparities exacerbate the time to cancer treatment and ultimately mortality, due to long travel times, low availability of clinical trials, and additional health burdens, an issue that is well observed in the state of Indiana^18^. Thus, additional research is clearly needed among US populations to delineate and improve the time to cancer diagnosis and treatment for ED-suspected cancer patients.

It has been demonstrated that emergent presentations of cancer are associated with lower curative rates and treatments, even when compared to cancers diagnosed “electively” (even at the same stage)^19^. The present data in Fig. 2, together with prior findings support the inference that patients who have a diagnosis of cancer temporally connected with an ED visit, suffer from a disparity in their odds of survival. From a health services standpoint, potentially modifiable causes for this disparity include lack of access to primary medical care and cancer screening before diagnosis, increased rate of tobacco and alcohol use, worsened uncontrolled comorbidities at the time of diagnosis, and lack of access to specialty cancer care after diagnosis. Thus, additional work should focus on reducing the time to cancer diagnosis as expedited diagnosis of symptomatic cancer likely benefits patients’ survival and improved quality of life as demonstrated in a systematic review of over 200 studies^20^.

This work employed a state-wide assessment of cancer diagnoses in the state of Indiana. From this, numerous patient factors appear to contribute to the observed mortality and worse outcomes, namely race and socioeconomic status. Meanwhile, factors such as age and sex appear to have little relationship between the association of diagnosis and mortality. This is compared to other works where older age (≥ 85 years old) are 2.5 times more likely to present with an emergent diagnosis of cancer, when compared to a 65–74-year-old cohort^21^. Those authors conclude that cancer and age are likely to reflect disease specific factors. Further, our work we excluded pediatric patients (< 18 years old), and it is well known that more than half of patients that present with de novo cancer diagnoses in the ED are emergently diagnosed^22^.

What limited evidence exists from data obtained in the United States, has demonstrated associations between socioeconomic status and the diagnosis of cancer as an emergency. African Americans in one study had increased odds of emergently diagnosed colorectal cancer (AOR 1.5, 95% CI 1.38–1.63) as compared to a similar white cohort^23^. This disparity not only exists among the diagnosis of cancer but in the primary and secondary preventions among lower SES populations. Colorectal screening, despite its efficacy and recommendations, has been shown to be low among African Americans and those with low SES, demonstrating an opportunity or intervening on this high risk population in the ED^24^. Furthermore, the low SES population have inequities that result in poor lifestyle choices, some of which (smoking status, diet, physical activity) are preventable and modifiable if this population had access to equitable opportunities. Cancer mortality among this population has an association between mortality and modifiable risk factors, again at current date are not routinely performed in the ED^25^. Blacks are diagnosed with breast and lung cancer in the cases versus controls and their outcomes appear to be worse. We speculate that blacks are more dependent upon EDs than whites for unscheduled, emergent care, and thus are more likely to present to an ED emergently for their undiagnosed malignancy^26^. Similar phenomenon can be applied to whites with low SES, where patients of low SES are more reliant on the ED for their care and are more likely to present emergently with their undiagnosed cancer^27,28^. These data suggest race and socioeconomic status are more important than other factors, such as comorbidities, since the CCI was equal. While not examined in this large data analyses, there is growing evidence that African Americans have more aggressive tumor biology, such as in several breast cancer studies, which can be speculated that this also contributes to these patients presenting more emergently for their undiagnosed cancer^29^.

Of the cancers that have proven screenings that demonstrate success, lung and colorectal cancers do markedly worse when associated with an ED-visit, 54% vs 18% mortality for lung cancer (*P* < 0.0001), and 26.4% vs 9% for colorectal cancer (*P* < 0.0001). Breast cancer also has a successful screening modality, namely mammography, and suffers from similar poor outcomes when associated with an ED visit, 11.9% mortality vs 3.4%, ED to non-ED associated^30^. Prostate cancer screening is controversial and recommendations vary by organization and country, regardless, a screening modality is available and those diagnosed with prostate cancer associated with an ED visit have higher mortality than those that don’t (13.2% vs 3.5%)^31^. Lastly, cervical cancer also is frequently screened for as outpatients and 22% of those seen in the ED were dead, versus 7.4% of those not seen in the ED. This evidence is supported by known disparities in cancer screening, with minority patients experiencing greater delays in evaluation and screening for cancer, leading to suboptimal treatment among those patients subsequently diagnosed with cancer^32^.

Previous research has called for both improving the outcomes of patients that are diagnosed with cancer through an emergent presentation, as well as helping to reduce the burden of emergent diagnoses by improving cancer screening^6,33^. This is likely a systems issue, but plausible future steps is utilizing the ED space for more than just emergent care. Average length of stays in ED has multiple variables that impact exact time frame which patients sit in the ED, but in one paper an average a patient can expect a wait of 4 h^34^. As the trend of increasing length of stay continues to increase nationwide, EDs are experiencing the unfortunate phenomena of ED crowding which has been well demonstrated to be associated with increased hospital death^35^. We propose future work in developing interventions for this at risk population while in the ED, similar to what has been performed for rapid hepatitis screening and cervical cancer screening in urban EDs, demonstrating proof of concept and utilizing the ED space for more than emergent care^36,37^. Similar revolutionary changes in ED workflow for improving the overall health of ED patients has been adopted with universal HIV screening in the ED, as well as universal suicide screening in EDs^38,39^. Intervening on this population is challenging but supported by a Cochrane Review, guaiac fecal immunochemical test (FIT) can reduce colorectal mortality by 15%, and providing appropriately chosen patients with home use FIT tests through primary care has improved screening rates, sustaining a screening rate of 75%^40,41^. Currently screening routinely for cancer does not occur in the emergency setting, but for many vulnerable patients (uninsured, lower SES, racial minorities), the emergency room serves as the only opportunity for routine care and we should begin to explore alternative strategies to maximally improve the care of these patients^42^. Removing the barriers to cancer screening, such as providing patients with FIT cards prior to discharge may represent an opportunity to increase adherence to CRC screening and reduce the burden of emergently diagnosed CRC^43^. Novel approaches need be undertaken at the systems and health policy level to address the disparities that are well demonstrated among ED-associated cancer diagnoses.

### Limitations

There are limitations to this study, namely the retrospective methodology to obtaining the administrative data. A diagnosis of cancer, while suspected in the ED, usually occurs weeks to months after the actual ED encounter, and thus confirming linkage from a suspected ED visit and a diagnosis is challenging. Further, patient stage and tumor specific biology are not included on the ICD-coded diagnosis and thus knowing the stage and extent of disease is not available without retrospective chart review. Additionally, survivor bias is present, because in order to be included in this study, one must have an ICD-coded cancer diagnosis. Regardless, we made an inference that being diagnosed with cancer within 6 months of a recorded ED visit, meant those ED physicians had an opportunity to diagnose asymptomatic cancer, if that ED visit wasn’t directly related to the presentation of emergent cancer diagnosis. The relationship between the ED visit and the diagnosis of cancer are unclear, due to lack of detailed ED visit information as well as most patients that are discharged from the ED have symptom-based discharge diagnoses as opposed to a definitive discharge diagnosis^44^. No detailed ED visit was curated from this study and thus linking the ED-diagnosis to the ultimate cancer diagnosis is not feasible in this study.

No knowledge is known about previous screenings and primary care follow up was available in this study, thus no inference can be made to know whether or not screening may have reduced the likelihood of the found associations. The lack of follow-up knowledge means we can’t examine the relationship between mortality and inadequate access to expert care, but the correction for clustering on logistic analyses suggests this is not just a result of location. Ultimately, it is difficult to determine if the observed outcome differences primarily reflect lead-time bias, health care disparities, or other factors, but comparing to the limited available data primarily from Europe, these data clearly present a concerning trend for patients with a new diagnosis of cancer. Unlike Europe which relies on the general practitioner to help coordinate cancer workups, cancer care-pathways are ill-defined and understudied. Thus, the outcomes of this present study may be very different in Europe and in other health care settings.

Further limitations include those cancers that are so advanced that tissue biopsy is not obtained, or patients prefer to not seek treatment. Lastly, pediatric cancers and their association with being emergently diagnosed in the ED were not explored in this study. No pediatric cancers have preventable or modifiable risk factors, and as such the goal of this work is to find interventions on cancers that can be prevented with lifestyle modification (smoking cessation, weight loss), or caught earlier with age-appropriate screening.

## Conclusions

Patients diagnosed with cancer with an associated ED visit within 6 months prior to their ICD-code cancer diagnosis are associated with poor outcomes, specifically increased mortality, as compared to a cohort that did not have an ED visit within 6 months prior to their diagnosis. Lung, breast, and colorectal are the most frequently ED-associated cancer diagnoses, with upwards of 50% mortality as compared to non-ED associated cancers. Further, there are associated racial and socioeconomic disparities among those diagnosed, both in cancer type and frequency, and mortality. These data are among the first to our knowledge that describe patients in the United States, among a statewide database. They further suggest that ED-associated cancer diagnoses offers an opportunity for additional research to understand the associations between diagnoses and socioeconomic and racial disparities. Given existing literature is limited to retrospective, database analyses, future work should be aimed at prospective studies as to guide future interventions to help reduce the disparities and reduce the mortality among ED-associated cancer diagnoses.

## Data Availability

All data generated or analyzed during this study are included in this published article (and its Supplementary information files).

## Abbreviations

ED: Emergency department
CCI: Charlson Comorbidity Index
